# Biofunctional few-layer metal dichalcogenides and related heterostructures produced by direct aqueous exfoliation using phospholipids†

**DOI:** 10.1039/c9ra07764b

**Published:** 2019-11-13

**Authors:** Aled T. Williams, Roberto Donno, Nicola Tirelli, Robert A. W. Dryfe

**Affiliations:** School of Chemistry, University of Manchester Oxford Road Manchester M13 9PL UK robert.dryfe@manchester.ac.uk; Laboratory of Polymers and Biomaterials, Fondazione Istituto Italiano di Tecnologia Via Morego, 30 Genoa Italy; National Graphene Institute, University of Manchester Oxford Road Manchester M13 9PL UK

## Abstract

We report a novel, inexpensive and green method for preparing aqueous dispersions of various biofunctional transition-metal dichalcogenides (MoS_2_, WS_2_, TiS_2_ and MoSe_2_) and their related heterostructures directly *via* ultrasonic exfoliation mediated by the presence of phospholipids. The dispersions predominantly consist of few-layer flakes coated with 1,2-dioleoyl-*sn-glycero*-3-phosphocholine (DOPC), as confirmed by Raman, photoluminescence and X-ray photoelectron spectroscopies. The phospholipid coating renders the flakes biofunctional, which coupled with the unique properties of transition-metal dichalcogenides and their heterostructures, suggests this method will have great potential in biological applications.

## Abstract 2

We report a method for preparing aqueous dispersions of biofunctional transition-metal dichalcogenides (MoS_2_, WS_2_, TiS_2_ and MoSe_2_) and their related heterostructures directly *via* ultrasonic exfoliation mediated by the presence of phospholipids.

## Graphical abstract

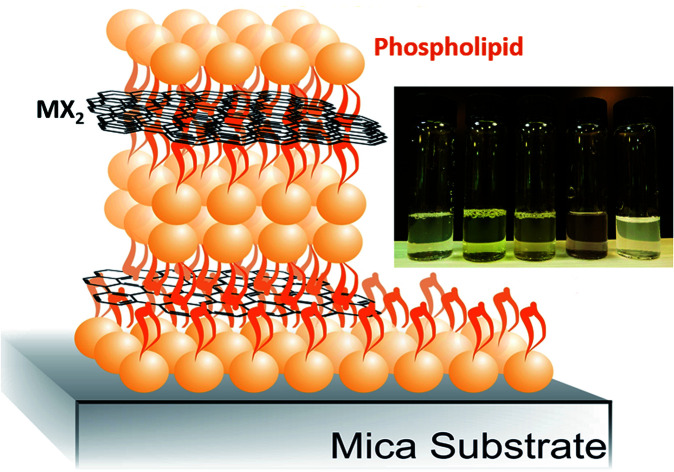

Transition-metal dichalcogenides (TMDCs) and other species of layered inorganic compounds are currently attracting much interest as the next generation of two-dimensional (2D) materials beyond graphene.^1^ These materials, notably MoS_2_, WS_2_ and hexagonal boron nitride (hBN), have already found applications in fields such as optoelectronics and energy storage due to their unique physical properties.^2–6^ Structurally, TMDCs are 2D layers of transition metal atoms, M, each covalently bound to two chalcogen atoms, X, and held together by van der Waals forces. The combination of metal and chalcogen influences the electronic properties of the material, for example TiS_2_ is a semimetal whilst MoS_2_ is a semiconductor in its naturally occurring, 2H, phase.^7^ In addition to MX_2_ compounds, similar structures are also embodied by hBN, Bi_2_Te_3_, transition-metal oxides, silicene, germanene and phosphorene (exfoliated black phosphorus) providing a range of new 2D materials for exploitation based on their individual attributes.^1,8^ There is also growing interest in hetero-structures formed from these materials, *i.e.* stacked combinations of different 2D species, offering further diversity in the properties that can be harvested from these layered materials.^2,9–11^

Solution processing of 2D materials is essential for industrial applications^12^ where the time-intensive and costly approaches of micromechanical cleavage and chemical vapour deposition hinder the commercial viability of some technologies. Several liquid-phase exfoliation methods have been reported over recent years, the majority of which have been based on earlier methods applied to the exfoliation of graphite.^13,14^ Notably, direct sonication of bulk powders in high boiling-point (HBP) solvents such as *N*-methyl-2-pyrrolidone (NMP) has been shown to produce monolayer to few-layer (layer number, *n* ≤ 3) dispersions of TMDCs and other layered inorganic materials with reasonable stabilities and concentrations (up to 40 mg mL^−1^).^15,16^ However, direct sonication using HBP solvents raises obvious economic, environmental and safety concerns and furthermore, HBP solvent residues in thin films fabricated from such dispersions can be difficult to remove, which can be detrimental to subsequent applications in electronics.^17^ Alternative preparation techniques, such as chemical and electrochemical Li-intercalation^18–20^ and various surface functionalization strategies using organic molecules,^19,21^ often involve multiple steps and undesirable reaction conditions.

Aqueous exfoliation methods can address many of these issues. However, successful liquid-phase exfoliation requires that the enthalpy of mixing per unit volume associated with the dispersal of the 2D flakes is close to zero, and it has been shown that for TMDCs^22^ optimal solvents are characterized by surface tensions in the region of 40 mJ m^−2^, with water falling outside this range at 72.75 mJ m^−2^ (at 20 °C).^23^ However, aqueous dispersions of hydrophobic flakes can be stabilized electrostatically or sterically, and a number of recent reports detail the use of surfactants or polymers in facilitating the aqueous exfoliation of layered inorganic materials *via* the application of ultrasonic energy.^24–28^ Herein, we report on a new exfoliation method, where phospholipids are used as electrostatic stabilizing agents in preparing aqueous dispersions of TMDCs directly *via* sonication of the bulk materials. Phospholipids have the additional advantage of imparting bio-compatibility to the 2D materials. We also demonstrate that the mixing and subsequent sonication of these dispersions result in the formation of hetero-structured materials, whose optoelectronic characteristics can be modified by the respective ratios of the parent materials.

Briefly, our method involves sonicating MX_2_ powders in ultra-pure water containing phospholipid for 12 h, followed by a centrifugation to purify the dispersions from large aggregates (full details can be found in the ESI†). We have previously described how this procedure can be applied to graphene exfoliation,^29^ where we have shown that the amount of 2D-material dispersed was dependent on the phospholipid concentration and the fluidity of the phospholipid aliphatic chains. Herein, we report that the same method can be used to produce meta-stable aqueous dispersions for each TMDC tested, namely MoS_2_, WS_2_, TiS_2_ and MoSe_2_, as well as hBN. For the dispersions characterised in this study, MX_2_ and hBN powders were sonicated in ultra-pure water with 1,2-dioleoyl-*sn-glycero*-3-phosphocholine (DOPC) at a concentration of 0.2 mg mL^−1^, resulting in dispersed concentrations in the range of 0.01–0.1 mg mL^−1^, depending on the 2D material used. DOPC was selected as the lipid of choice on the basis of our graphene dispersion work,^29^ as the fluidity of this phospholipid chain was shown to impart a good level of dispersion stability compared to other phospholipids. Thermogravimetric analysis (TGA) performed on freeze-dried dispersions of MoS_2_, WS_2_ and TiS_2_ suggest that the method typically produces fresh dispersions containing 10–20% weight of dispersed 2D-material (refer to Fig. S1 in the ESI†), comparable to that observed for graphene dispersions produced by the same method.^29^

Ultraviolet-visible (UV-vis) spectroscopy confirmed the presence of dispersed few-layer MX_2_ species, with the following characteristic major absorption peaks: 393, 450, 608 and 665 nm for MoS_2_;^3,30,31^ 412, 697 and 805 nm for MoSe_2_;^3,32^ 593 nm for TiS_2_ ^33^ and 624 nm for WS_2_.^3,30^ No absorption peaks could be discerned from the Rayleigh scattering in the spectrum for hBN. The spectra are presented in Fig. 1A together with photographs of the dispersions.

**Fig. 1:**
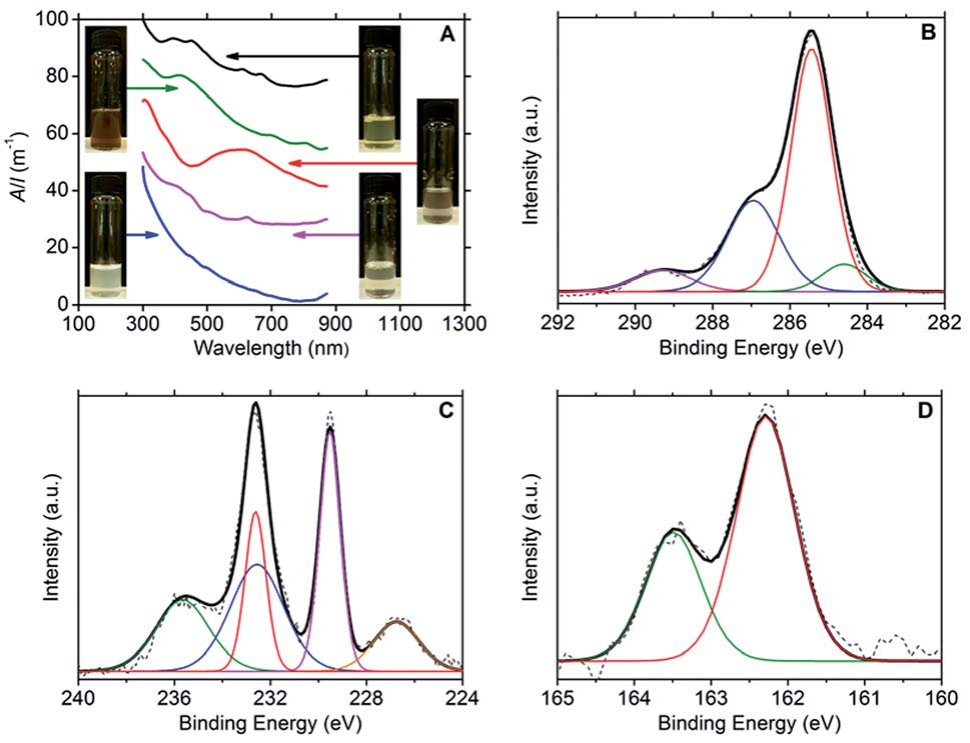
(A) UV-vis absorbance spectra of the aqueous DOPC/2D-material dispersions (from top to bottom): MoS_2_ (black line), MoSe_2_ (green line), TiS_2_ (red line), WS_2_ (purple line) and hBN (blue line). The stacked spectra are in proportion to each other. (B) C1s XPS spectrum of a DOPC/MoS_2_ dispersion drop cast onto Si/SiO_2_ substrate. In the fitting, the green line corresponds to the C–C sp^2^ peak, the red line to the C–C sp^3^ peak, the blue line to the C–O/C–N peak and the purple line to the O–C=O peak. (C) Mo3d XPS spectrum of a DOPC/MoS_2_ dispersion drop cast onto Si/SiO_2_ substrate. In the fitting, the orange line corresponds to the S2s peak, the purple line to the Mo^4+^3d_5/2_ peak, the red line to the Mo^4+^3d_3/2_ peak, the blue line to the Mo^6+^3d_5/2_ peak and the green line to the Mo^6+^3d_3/2_ peak. (D) S2p XPS spectrum of a DOPC/MoS_2_ dispersion drop cast onto Si/SiO_2_ substrate. The fitting is for the S2p doublet, with the red line depicting the S2p_3/2_ peak and the green line the S2p_1/2_ peak.

Hydrodynamic diameter values obtained *via* dynamic light scattering (DLS) measurements are presented in Table 1, providing rough estimates (between tens and hundreds of nm, due to the approximations in the technique) of colloid size which were found to be comparable to those observed for graphene dispersions produced by the same method.^29^ The dispersed TMDCs were characterized by negative *ζ* potential values at neutral pH (see Table 1), although smaller in magnitude to those observed for the graphene dispersions (−34 mV). Shorter-term stability is expected as a result of this observation and this is confirmed by comparison of the time-dependent reductions in optical density presented in Table 1. It is worth noting that the TiS_2_ dispersions, approximately 12 h after preparation, produce a strong sulphurous odour: it is known that this material is prone to oxidation in aqueous solution, most likely *via* the following reaction:^34^TiS_2_ + *x*H_2_O → TiS_2−*x*_O_*x*_ + *x*H_2_S

**Table 1:** Summary of *ζ* potential and DLS hydrodynamic diameter values (*D*) as measured for various 2D-material/DOPC aqueous dispersions, as well as stability data in the form of reduction in optical density *versus* day 0 (ODR)

Material	ζ (mV)	*D* (nm)	ODR day 10^a^	ODR day 20^a^	ODR day 30^a^	ODR day 50^a^
MoS_2_	−20	142	8%	16%	32%	71%
MoSe_2_	−28	164	14%	23%	34%	87%
TiS_2_	−19	495	56%	69%	81%	92%
WS_2_	−15	255	14%	29%	51%	85%
hBN	−23	295	70%	74%	80%	85%
Graphene	−34	190	—	11%^b^	25%^b^	43%^b^

a Optical density measured at *A*_608_ (MoS_2_), *A*_805_ (MoSe_2_), *A*_565_ (TiS_2_), *A*_624_ (WS_2_), *A*_300_ (hBN) and *A*_660_ (graphene), where subscripts are wavelengths in nanometres. Graphene data at *A*_660_ taken from ref. 29.

b Optical density data for graphene measured at *A*_660_ at time points: 22, 41 and 56 days.

As the hBN dispersions were found to precipitate at a significantly faster rate to the other 2D materials, no further characterisation work was conducted on hBN dispersions as part of this study.

X-ray photoelectron spectroscopy (XPS) was used to confirm the presence of DOPC and MX_2_ in the dispersions; refer to Fig. 1B–D (and Fig. S2–S4 in the ESI†). For DOPC, the C1s spectrum for all samples (dispersions of MoS_2_, MoSe_2_, WS_2_ and TiS_2_) showed peaks representatives of DOPC at average binding energies of 284.5 eV (C–C sp^2^), 285.3 eV (C–C sp^3^), 286.9 eV (combined signal for C–O and C–N) and 289.2 eV (O–C=O).^29^ Furthermore, the phosphorous and nitrogen peaks were found to be present at similar percentage atomic concentrations appearing at average binding energies of 134.0 eV and 134.9 eV (P2p_3/2_ and P2p_1/2_) and 402.9 eV respectively (N1s). For MoS_2_, the Mo3d_3/2_ and Mo3d_5/2_ doublet peaks were located at 232.6 eV and 229.5 eV respectively, with the S2s peak at 226.7 eV and the S2p doublet at 163.5 eV and 162.3 eV (S2p_1/2_ and S2p_3/2_). These binding energies are indicative of the expected Mo^4+^ and S^2−^ chemical states.^35–38^ However, the presence of a peak at 235.6 eV is indicative of the Mo3d_3/2_ doublet peak for Mo^6+^, and fitting reveals the complementary Mo3d_5/2_ peak at 232.5 eV, which strongly suggests that MoO_3_ is present at approximately the same concentration as MoS_2_ (percentage atomic concentrations of 47% Mo^4+^ and 53% Mo^6+^).^37–40^ The Mo^6+^ state was more heavily detected for the MoSe_2_ dispersions (percentage atomic concentrations of 11% Mo^4+^ and 89% Mo^6+^). The Mo3d_3/2_ and Mo3d_5/2_ doublet peaks for Mo^6+^ appear at 236.1 eV and 232.9 eV respectively, the same doublet peaks for Mo^4+^ being found at lower intensities at 232.5 eV and 229.4 eV, with the Se3d_3/2_ and Se3d_5/2_ doublet peaks appearing at 56.6 eV and 55.8 eV.^36^ For WS_2_, two chemical states were also detected for tungsten, W^4+^ and W^6+^. The W4f_5/2_ and W4f_7/2_ doublet peaks expected for WS_2_ are observed at 34.6 eV and 32.7 eV with the S2p doublet peaks appearing at 163.4 eV and 162.2 eV (S2p_1/2_ and S2p_3/2_). The W4f_5/2_ and W4f_7/2_ doublet peaks characteristic of the W^6+^ chemical state are seen at 38.1 eV and 36.0 eV, and represent the dominant binding energies of the W4f signal (percentage atomic concentrations of 12% W^4+^ and 88% W^6+^),^35,36,41^ which coupled with the weak S2p signal suggests that the samples contained significant amounts of WO_3_. For TiS_2_, the Ti2p_1/2_ and Ti2p_3/2_ doublet peaks appear at 464.8 eV and 459.1 eV respectively, with the S2p doublet peaks appearing at 165.2 eV and 163.9 eV (S2p_1/2_ and S2p_3/2_). These binding energies are indicative of the expected Ti^4+^ and S^2−^ chemical states.^42,43^ However, as with that observed for the WS_2_ sample, a weak S2p signal intensity was observed when compared to the Ti2p signal intensity. This observation, in conjunction with the dispersion stability data, suggests that oxidised titanium is also present in significant quantity. The partial oxidation of the TMDCs, detected by the XPS, could explain the lower long-term stability of the DOPC-stabilised dispersions relative to the graphene sample (see Table 1). Consistent with this, it is noted that the TiS_2_ sample has the lowest level of dispersion stability.

Raman spectroscopy was used to further characterise the DOPC/MX_2_ dispersions and to confirm the presence of few-layer flakes. Analysis was performed on drop-cast samples using Si/SiO_2_ substrates. For MoS_2_, spectra diagnostic of few-layer flakes predominated with the A_1g_ and E^1^_2g_ typically appearing at 406 cm^−1^ and 382 cm^−1^ respectively, separated by 24 cm^−1^, yielding maximum photoluminescent (PL) emission at 675 nm (1.84 eV), which is consistent with literature values for few-layer MoS_2_.^44–49^ Similarly, the spectra measured for the WS_2_ samples were found to be representative of few-layer material,^47–52^ with A_1g_ and E^1^_2g_ appearing at 419 cm^−1^ and 351 cm^−1^ respectively, separated by 68 cm^−1^ and yielding maximum photoluminescent (PL) emission at 633 nm (1.96 eV). Few-layer flakes were also detected for MoSe_2_ and TiS_2_. For MoSe_2_, this was confirmed by the following representative peaks: 351 cm^−1^ (B^1^_2g_), 285 cm^−1^ (E^1^_2g_) and 241 cm^−1^ (A_1g_)^48,49,53^ and for TiS_2_: 377 cm^−1^ (A_2u_), 333 cm^−1^ (A_1g_) and 233 cm^−1^ (E_g_),^33,49,54^ with the A_1g_ : A_2u_ peak intensity ratio at approximately 2.0 (compare to a value of 1.6 measured for the bulk powder starting-material).^33^ Weaker PL emissions were observed for the samples derived from the MoSe_2_ and TiS_2_ dispersions, as compared to those from MoS_2_ and WS_2_ dispersions. Fig. 2A and S5–S8 in the ESI† give typical examples of the Raman and PL spectra measured.

**Fig. 2:**
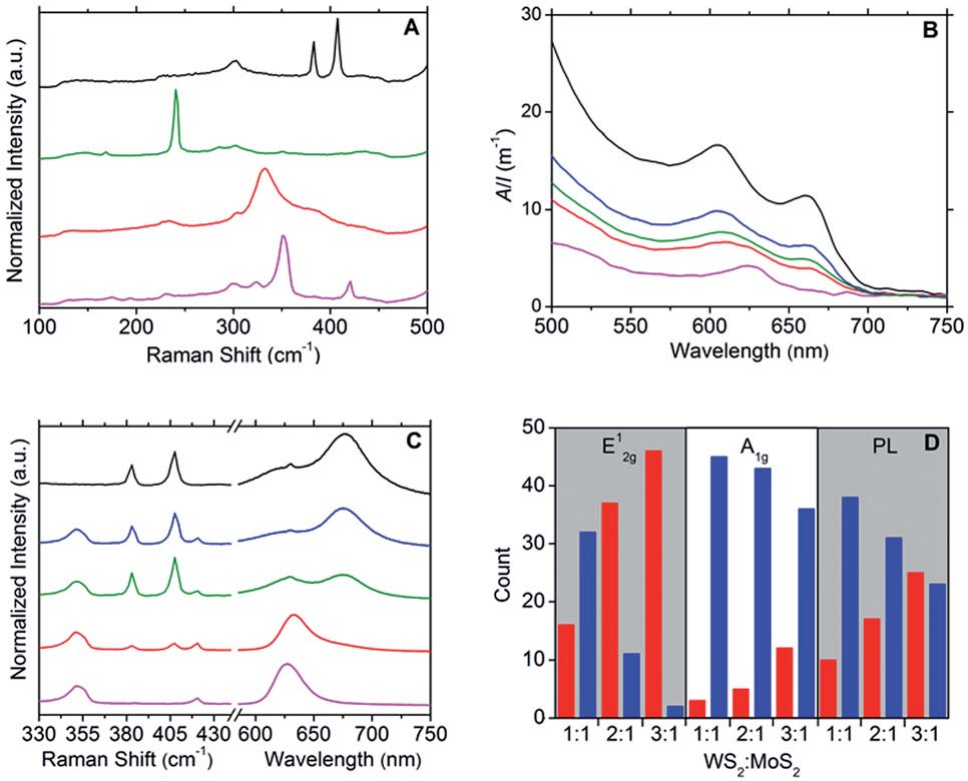
(A) Raman spectra (532 nm excitation) of the DOPC/MX_2_ dispersions drop cast onto Si/SiO_2_ substrates, corresponding (from top to bottom) to MoS_2_ (black line), MoSe_2_ (green line), TiS_2_ (red line) and WS_2_ (purple line). (B) UV-vis spectra of the following aqueous DOPC/2D-material dispersions (from top to bottom): MoS_2_ (black line), 1 : 1 WS_2_ : MoS_2_ heterostructures (blue line), 2 : 1 WS_2_ : MoS_2_ heterostructures (green line), 3 : 1 WS_2_ : MoS_2_ heterostructures (red line) and WS_2_ (purple line). (C) Typical Raman and PL spectra (532 nm excitation) measured for the following DOPC/2D-material dispersions drop cast onto Si/SiO_2_ substrates (from top to bottom): MoS_2_ (black line), 1 : 1 WS_2_ : MoS_2_ heterostructures (blue line), 2 : 1 WS_2_ : MoS_2_ heterostructures (green line), 3 : 1 WS_2_ : MoS_2_ heterostructures (red line) and WS_2_ (purple line). (D) Histograms showing the relative proportions of dominant peak intensities in the Raman (E^1^_2g_ and A_1g_) and PL spectra (*λ*_max_) of aqueous DOPC-mediated dispersions of 1 : 1, 2 : 1 and 3 : 1 WS_2_ : MoS_2_ heterostructures. Instances where the peak intensities arising from WS_2_ are greater than those from MoS_2_ are binned on the left-hand side (red columns) with the converse case binned on the right-hand side (blue columns); a bin size of 48 measurements (at different sample spots) was used in all instances.

Finally, atomic force microscopy (AFM) performed on drop-cast samples of DOPC/MoS_2_ dispersions (clean mica substrates were used) showed structures similar to those observed for graphene dispersions produced by the same method,^29^ with an average height of 40 nm. The AFM height images showed objects characterized by “steps” with a height of 5 nm (or multiple of 5 nm), which is a thickness comparable to that of a DOPC bilayer;^55–58^ refer to Fig. S9 in the ESI† for further details.

Supplemental to the novel properties offered by true 2D materials and their few-layer counterparts, mixed van der Waals stacking of different 2D materials to form heterostructures, endow few-layer materials with scope to impart bespoke properties.^2,9–11,38,59^ In addition to our method detailed above for the production of aqueous DOPC/MX_2_ dispersions, we also present a fast and single-step method to prepare aqueous DOPC/heterostructure dispersions. Briefly, the method involves mixing the desired parent DOPC/MX_2_ dispersions at specific volume ratios, after which the mixtures are sonicated for 10 minutes to facilitate the de-stacking and subsequent re-stacking of the parent layers into hetero-layered stacks. The method was used to prepare aqueous DOPC/WS_2_ : MoS_2_ dispersions, where the resulting optoelectronic properties were found to be tuneable to the relative quantities of the parent dispersions used. UV-vis spectroscopy (Fig. 2B) was used as a simple screening tool for the selection of appropriate volume ratios of DOPC/WS_2_ (*x*) and DOPC/MoS_2_ (*y*) dispersions to produce heterostructure dispersions with desired optoelectronic properties. For example, the UV-vis spectrum of the DOPC/WS_2_ : MoS_2_ dispersion prepared using the *x* : *y* ratio at 2 : 1 was found to be intermediate in character between those of the parent dispersions, with flakes (deposited on Si/SiO_2_ substrates) also displaying intermediate character with regard to Raman and PL response; refer to Fig. 2C. Further illustration of this optoelectronic tuning is shown *via* the histograms presented in Fig. 2D, which are based on Raman and PL data measured from a total of 48 flakes per DOPC/WS_2_ : MoS_2_ dispersion (*x* : *y* ratio at 1 : 1, 2 : 1 and 3 : 1). The measured flakes were categorised into two conditions: one where the Raman (A_1g_ and E^1^_2g_) and PL (*λ*_max_) intensities were greater for peaks emanating from WS_2_ (approximately 419 cm^−1^ for A_1g_, 350 cm^−1^ for E^1^_2g_ and 633 nm for PL) and the other where the intensities were greater for peaks emanating from MoS_2_ (approximately 407 cm^−1^ for A_1g_, 383 cm^−1^ for E^1^_2g_ and 675 nm for PL). As expected, the intensities of the peaks arising from WS_2_ become greater as *x* increases and as such, the findings show that manipulation of the *x* : *y* volume ratio allows for the tuning of the predominant optoelectronic properties of heterostructure flakes.

## Conclusions

In summary, a simple method has been presented to produce aqueous dispersions of phospholipid-coated few-layer transition-metal dichalcogenides (MoS_2_, WS_2_, TiS_2_ and MoSe_2_) *via* single-step exfoliation of the bulk starting materials in water. Furthermore, the resulting dispersions can be subsequently mixed at pre-defined volume ratios to produce heterostructured dispersions thereby allowing for the tuning of the optoelectronic properties. The phospholipid coating renders the flakes biofunctional, which coupled with the unique properties of transition-metal dichalcogenides, indicates the great potential of this method for use in biological applications, for example *in vivo* sensing or drug transport.

## Conflicts of interest

The authors declare no conflict of interest.

## Supplementary Material

RA-009-C9RA07764B-s001

## References

[cit1] Butler S. Z., Hollen S. M., Cao L. Y., Cui Y., Gupta J. A., Gutierrez H. R., Heinz T. F., Hong S. S., Huang J. X., Ismach A. F., Johnston-Halperin E., Kuno M., Plashnitsa V. V., Robinson R. D., Ruoff R. S., Salahuddin S., Shan J., Shi L., Spencer M. G., Terrones M., Windl W., Goldberger J. E. (2013). ACS Nano.

[cit2] Hong X. P., Kim J., Shi S. F., Zhang Y., Jin C. H., Sun Y. H., Tongay S., Wu J. Q., Zhang Y. F., Wang F. (2014). Nat. Nanotechnol..

[cit3] Wang Q. H., Kalantar-Zadeh K., Kis A., Coleman J. N., Strano M. S. (2012). Nat. Nanotechnol..

[cit4] Tsai M. L., Su S. H., Chang J. K., Tsai D. S., Chen C. H., Wu C. I., Li L. J., Chen L. J., He J. H. (2014). ACS Nano.

[cit5] Firmiano E. G. D., Rabelo A. C., Dalmaschio C. J., Pinheiro A. N., Pereira E. C., Schreiner W. H., Leite E. R. (2014). Adv. Energy Mater..

[cit6] Lee G. H., Yu Y. J., Cui X., Petrone N., Lee C. H., Choi M. S., Lee D. Y., Lee C., Yoo W. J., Watanabe K., Taniguchi T., Nuckolls C., Kim P., Hone J. (2013). ACS Nano.

[cit7] Chhowalla M., Shin H. S., Eda G., Li L. J., Loh K. P., Zhang H. (2013). Nat. Chem..

[cit8] Mas-Balleste R., Gomez-Navarro C., Gomez-Herrero J., Zamora F. (2011). Nanoscale.

[cit9] Huang C. M., Wu S. F., Sanchez A. M., Peters J. J. P., Beanland R., Ross J. S., Rivera P., Yao W., Cobden D. H., Xu X. D. (2014). Nat. Mater..

[cit10] Fang H., Battaglia C., Carraro C., Nemsak S., Ozdol B., Kang J. S., Bechtel H. A., Desai S. B., Kronast F., Unal A. A., Conti G., Conlon C., Palsson G. K., Martin M. C., Minor A. M., Fadley C. S., Yablonovitch E., Maboudian R., Javey A. (2014). Proc. Natl. Acad. Sci. U. S. A..

[cit11] Wang H., Liu F. C., Fu W., Fang Z. Y., Zhou W., Liu Z. (2014). Nanoscale.

[cit12] Finn D. J., Lotya M., Cunningham G., Smith R. J., McCloskey D., Donegan J. F., Coleman J. N. (2014). J. Mater. Chem. C.

[cit13] Hernandez Y., Nicolosi V., Lotya M., Blighe F. M., Sun Z. Y., De S., McGovern I. T., Holland B., Byrne M., Gun'ko Y. K., Boland J. J., Niraj P., Duesberg G., Krishnamurthy S., Goodhue R., Hutchison J., Scardaci V., Ferrari A. C., Coleman J. N. (2008). Nat. Nanotechnol..

[cit14] Khan U., O'Neill A., Lotya M., De S., Coleman J. N. (2010). Small.

[cit15] Coleman J. N., Lotya M., O'Neill A., Bergin S. D., King P. J., Khan U., Young K., Gaucher A., De S., Smith R. J., Shvets I. V., Arora S. K., Stanton G., Kim H. Y., Lee K., Kim G. T., Duesberg G. S., Hallam T., Boland J. J., Wang J. J., Donegan J. F., Grunlan J. C., Moriarty G., Shmeliov A., Nicholls R. J., Perkins J. M., Grieveson E. M., Theuwissen K., McComb D. W., Nellist P. D., Nicolosi V. (2011). Science.

[cit16] O'Neill A., Khan U., Coleman J. N. (2012). Chem. Mater..

[cit17] Hernandez T. C., Blanco A. C. F., Williams A. T., Velicky M., Patten H. V., Colina A., Dryfe R. A. W. (2015). Electroanalysis.

[cit18] Zheng J., Zhang H., Dong S. H., Liu Y. P., Nai C. T., Shin H. S., Jeong H. Y., Liu B., Loh K. P. (2014). Nat. Commun..

[cit19] Huang X., Zeng Z. Y., Zhang H. (2013). Chem. Soc. Rev..

[cit20] Gordon R. A., Yang D., Crozier E. D., Jiang D. T., Frindt R. F. (2002). Phys. Rev. B.

[cit21] Zhou L., He B. Z., Yang Y., He Y. G. (2014). RSC Adv..

[cit22] Cunningham G., Lotya M., Cucinotta C. S., Sanvito S., Bergin S. D., Menzel R., Shaffer M. S. P., Coleman J. N. (2012). ACS Nano.

[cit23] Vargaftik N. B., Volkov B. N., Voljak L. D. (1983). J. Phys. Chem. Ref. Data.

[cit24] Smith R. J., King P. J., Lotya M., Wirtz C., Khan U., De S., O'Neill A., Duesberg G. S., Grunlan J. C., Moriarty G., Chen J., Wang J. Z., Minett A. I., Nicolosi V., Coleman J. N. (2011). Adv. Mater..

[cit25] Yao Y. G., Tolentino L., Yang Z. Z., Song X. J., Zhang W., Chen Y. S., Wong C. P. (2013). Adv. Funct. Mater..

[cit26] Quinn M. D. J., Ho N. H., Notley S. M. (2013). ACS Appl. Mater. Interfaces.

[cit27] Guardia L., Paredes J. I., Rozada R., Villar-Rodil S., Martinez-Alonso A., Tascon J. M. D. (2014). RSC Adv..

[cit28] Gupta A., Arunachalam V., Vasudevan S. (2015). J. Phys. Chem. Lett..

[cit29] Williams A. T., Donno R., Tirelli N., Dryfe R. A. W. (2018). RSC Adv..

[cit30] Nguyen T. P., Sohn W., Oh J. H., Jang H. W., Kim S. Y. (2016). J. Phys. Chem. C.

[cit31] Bilgin I., Liu F., Vargas A., Winchester A., Man M. K. L., Upmanyu M., Dani K. M., Gupta G., Talapatra S., Mohite A. D., Kar S. (2015). ACS Nano.

[cit32] Berahim N., Amiri I. S., Anwar T., Azzuhri S. R., Mohd Nasir M. N. S., Zakaria R., Chong W. Y., Lai C. K., Lee S. H., Ahmad H., Ismail M. A., Yupapin P. (2019). Results Phys..

[cit33] Sherrell P. C., Sharda K., Grotta C., Ranalli J., Sokolikova M. S., Pesci F. M., Palczynski P., Bemmer V. L., Mattevi C. (2018). ACS Omega.

[cit34] Long E., O'Brien S., Lewis E. A., Prestat E., Downing C., Cucinotta C. S., Sanvito S., Haigh S. J., Nicolosi V. (2017). npj 2D Mater. Appl..

[cit35] Chiu M.-H., Zhang C., Shiu H.-W., Chuu C.-P., Chen C.-H., Chang C.-Y. S., Chen C.-H., Chou M.-Y., Shih C.-K., Li L.-J. (2015). Nat. Commun..

[cit36] Guo J., Shi Y., Bai X., Wang X., Ma T. (2015). J. Mater. Chem. A.

[cit37] Tai G., Zeng T., Yu J., Zhou J., You Y., Wang X., Wu H., Sun X., Hu T., Guo W. (2016). Nanoscale.

[cit38] Lin Z., Carvalho B. R., Kahn E., Lv R., Rao R., Terrones H., Pimenta M. A., Terrones M. (2016). 2D Materials.

[cit39] Nan H., Wang Z., Wang W., Liang Z., Lu Y., Chen Q., He D., Tan P., Miao F., Wang X., Wang J., Ni Z. (2014). ACS Nano.

[cit40] Madhavi V., Kondaiah P., Rayudu S. S., Hussain O. M., Uthanna S. (2013). Mater. Express.

[cit41] McCreary K. M., Hanbicki A. T., Jernigan G. G., Culbertson J. C., Jonker B. T. (2016). Sci. Rep..

[cit42] Lin C., Zhu X., Feng J., Wu C., Hu S., Peng J., Guo Y., Peng L., Zhao J., Huang J., Yang J., Xie Y. (2013). J. Am. Chem. Soc..

[cit43] Tonti D., Pettenkofer C., Jaegermann W. (2000). Electrochem. Solid-State Lett..

[cit44] Ganatra R., Zhang Q. (2014). ACS Nano.

[cit45] Mak K. F., Lee C., Hone J., Shan J., Heinz T. F. (2010). Phys. Rev. Lett..

[cit46] Splendiani A., Sun L., Zhang Y., Li T., Kim J., Chim C.-Y., Galli G., Wang F. (2010). Nano Lett..

[cit47] Bissett M. A., Hattle A. G., Marsden A. J., Kinloch I. A., Dryfe R. A. W. (2017). ACS Omega.

[cit48] Niu Y., Gonzalez-Abad S., Frisenda R., Marauhn P., Drüppel M., Gant P., Schmidt R., Taghavi S. N., Barcons D., Molina-Mendoza J. A., De Vasconcellos M. S., Bratschitsch R., Perez De Lara D., Rohlfing M., Castellanos-Gomez A. (2018). Nanomaterials.

[cit49] Bissett M., Worrall S., Kinloch I., Dryfe R. A. W. (2016). Electrochim. Acta.

[cit50] Zhao W., Ghorannevis Z., Chu L., Toh M., Kloc C., Tan P.-H., Eda G. (2013). ACS Nano.

[cit51] Berkdemir A., Gutiérrez H. R., Botello-Méndez A. R., Perea-López N., Elías A. L., Chia C.-I., Wang B., Crespi V. H., López-Urías F., Charlier J.-C., Terrones H., Terrones M. (2013). Sci. Rep..

[cit52] Bianco G. V., Losurdo M., Giangregorio M. M., Sacchetti A., Prete P., Lovergine N., Capezzuto P., Bruno G. (2015). RSC Adv..

[cit53] Tonndorf P., Schmidt R., Böttger P., Zhang X., Börner J., Liebig A., Albrecht M., Kloc C., Gordan O., Zahn D. R. T., Michaelis de Vasconcellos S., Bratschitsch R. (2013). Opt. Express.

[cit54] Dolui K., Sanvito S. (2016). Europhys. Lett..

[cit55] Hirtz M., Oikonomou A., Georgiou T., Fuchs H., Vijayaraghavan A. (2013). Nat. Commun..

[cit56] Wang S. T., Fukuto M., Yang L. (2008). Phys. Rev. E: Stat., Nonlinear, Soft Matter Phys..

[cit57] Dols-Perez A., Fumagalli L., Gomila G. (2014). Colloids Surf., B.

[cit58] Leonenko Z. V., Finot E., Ma H., Dahms T. E. S., Cramb D. T. (2004). Biophys. J..

[cit59] Liang L., Meunier V. (2014). Nanoscale.

