# Supplementary material for: Biofunctional few-layer metal dichalcogenides and related heterostructures produced by direct aqueous exfoliation using phospholipids
Source: RSC Adv. 2019 Nov 13;9(63):37061–6. doi: 10.1039/c9ra07764b (PMC9075593; doi:10.1039/c9ra07764b)
Supplement: RA-009-C9RA07764B-s001 [file RA-009-C9RA07764B-s001.pdf]

Electronic Supplementary Material (ESI) for RSC Advances.
This journal is © The Royal Society of Chemistry 2019

Supplementary Information

Biofunctional Few-layer Metal Dichalcogenides and Related Heterostructures Produced by Direct Aqueous Exfoliation Using Phospholipids

Aled T. Williams[a], Roberto Donno[b], Nicola Tirelli[b] and Robert A. W. Dryfe[a, c]

a. *School of Chemistry, University of Manchester, Oxford Road, Manchester, M13 9PL, UK.*

b. *Laboratory of Polymers and Biomaterials, Fondazione Istituto Italiano di Tecnologia, Via Morego, 30, Genoa, Italy.*

c. *National Graphene Institute, University of Manchester, Oxford Road, Manchester, M13 9PL, UK.*

Materials

All starting materials and solvents were used as received. MoS_2 (purum), WS_2 (2 µm, 99%), TiS_2 (-200 mesh, 99.9%), $MoSe_2$ (-325 mesh, 99.9%) and BN (1 µm, 98%) powders were purchased from Sigma-Aldrich. Graphite powder (grade 2369) was obtained from Graphexel Ltd (UK). 1,2-dioleoyl-*sn*-glycero-3-phosphocholine (DOPC) (≥ 99%) was purchased from Sigma-Aldrich and kept refrigerated.

Preparation of dispersions

In a typical experiment, DOPC was dispersed in 10 mL of ultra-pure water (18 MΩ cm, Milli-Q Direct 8, Millipore, USA) at a concentration of 0.2 mg mL^{-1} in a 20 mL glass vial. The inorganic material, in powder form, was sieved before use through a 500 µm pore-size mesh in order to remove large particles, and subsequently added to the phospholipid/water solution at a concentration of 5 mg mL^{-1}. The mixture was shaken and then sonicated in an ultrasonic bath for 12 hours (Elmasonic P, Elma, Germany. Frequency = 37 kHz, power equivalent = 40%, true power ≈ 34 W) at a constant bath temperature of 20 °C, after which the resulting dispersion was left standing for 24 hours to allow for the sedimentation of large aggregates. The supernatant was subsequently removed and centrifuged at 6000 rpm for 30 min, with the process being repeated once more to produce a dispersion. The amount of dispersed material was calculated by carefully separating the supernatants from the sediment after centrifugation. The volume of dispersion was measured and the undispersed mass was dried in a vacuum oven (Gallenkamp, UK) at 120 °C. For the preparation of DOPC/MX_2:MX_2 heterostructure dispersions, x and y volume ratios of the desired parent DOPC/MX_2 dispersions were mixed and subsequently sonicated for 10 minutes.

Thermogravimetric analysis (TGA)

15 mL aliquots of supernatants were freeze dried in a 50 mL Falcon tube (Fisherbrand, polypropylene) using a Genevac evaporator (EZ-2 series); the resulting solids were transferred to a cellulose filter, repeatedly washed with ultrapure water and allowed to dry in a vacuum oven at 120 °C for 24 hrs. TGA measurements were performed on a Q500 instrument (TA Instruments, USA) under a nitrogen atmosphere and at 10 °C min^{-1} using a temperature range of 20-1000 °C.

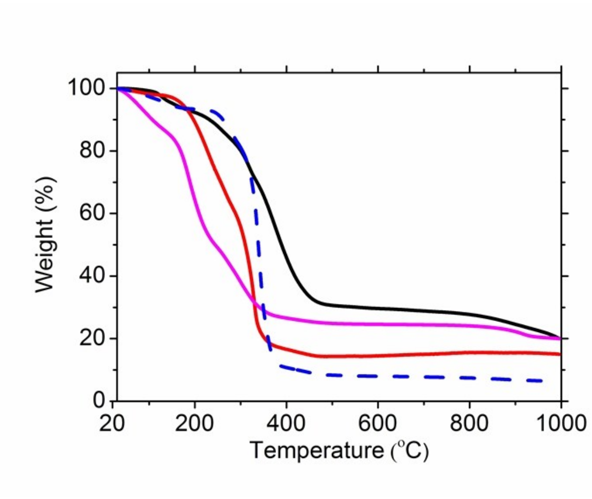

Figure S1: TGA of DOPC/MoS_2 dispersions (black solid line), DOPC/TiS_2 dispersions (red solid line), DOPC/WS_2 dispersions (purple solid line) and of pure DOPC (blue dashed line).

UV-Vis spectroscopy

UV-visible absorption spectra were recorded on a USB2000+UV-VIS fibre-optic spectrometer using a DH-2000-BAL deuterium-halogen light source (Ocean Optics).

Dynamic light scattering (DLS) and Zeta-potential measurements

A Malvern Zetasizer Nano ZS was operated in backscatter mode (173°), using a 633 nm HeNe laser. The 2D-material/DOPC dispersions were diluted in ultra-pure water (1:10 dilution) and equilibrated by the instrument to 25 °C for each measurement. The zeta potential was calculated by Malvern software using the Smoluchowski model.

X-ray photoelectron spectroscopy (XPS)

Samples were prepared by drop casting 100 µL of fresh dispersions onto 1 cm^2 Si/SiO_2 substrates (pre-cleaned by sonication in ethanol and acetone for 10 min), which were allowed to dry overnight. Spectra were measured using an AXIS Nova (Kratos Analytical, UK) with a monochromatic AlKα source operated at 225 W (15 kV and 15 mA). Analysis was performed using CasaXPS software, utilizing Shirley background subtraction and the appropriate relative sensitivity factors.

XPS: $MoSe_2$

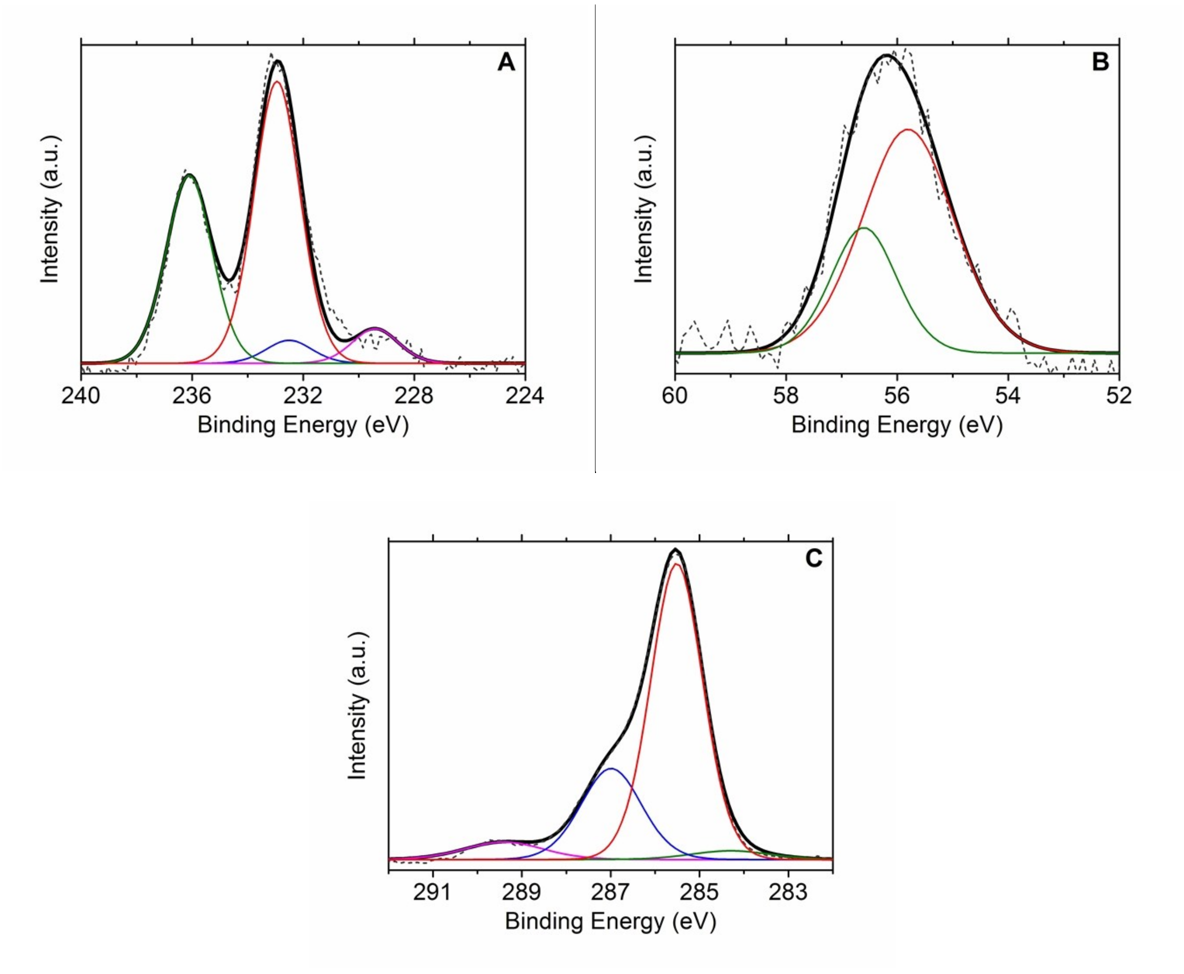

Fig. S2: **(A)** Mo3d XPS spectrum of a DOPC/$MoSe_2$ dispersion drop cast onto Si/SiO_2 substrate. In the fitting, the purple line corresponds to the $Mo^{4+}3d_{5/2}$ peak, the blue line to the $Mo^{4+}3d_{3/2}$ peak, the red line to the $Mo^{6+}3d_{5/2}$ peak and the green line to the $Mo^{6+}3d_{3/2}$ peak. **(B)** Se3d XPS spectrum of a DOPC/$MoSe_2$ dispersion drop cast onto Si/SiO_2 substrate. The fitting is for the Se3d doublet, with the red line depicting the $Se3d_{5/2}$ peak and the green line the $Se3d_{3/2}$ peak. **(C)** C1s XPS spectrum of a DOPC/$MoSe_2$ dispersion drop cast onto Si/SiO_2 substrate. In the fitting, the green line corresponds to the C-C sp^2 peak, the red line to the C-C sp^3 peak, the blue line to the C-O/C-N peak and the purple line to the O-C=O peak.

XPS: WS_2

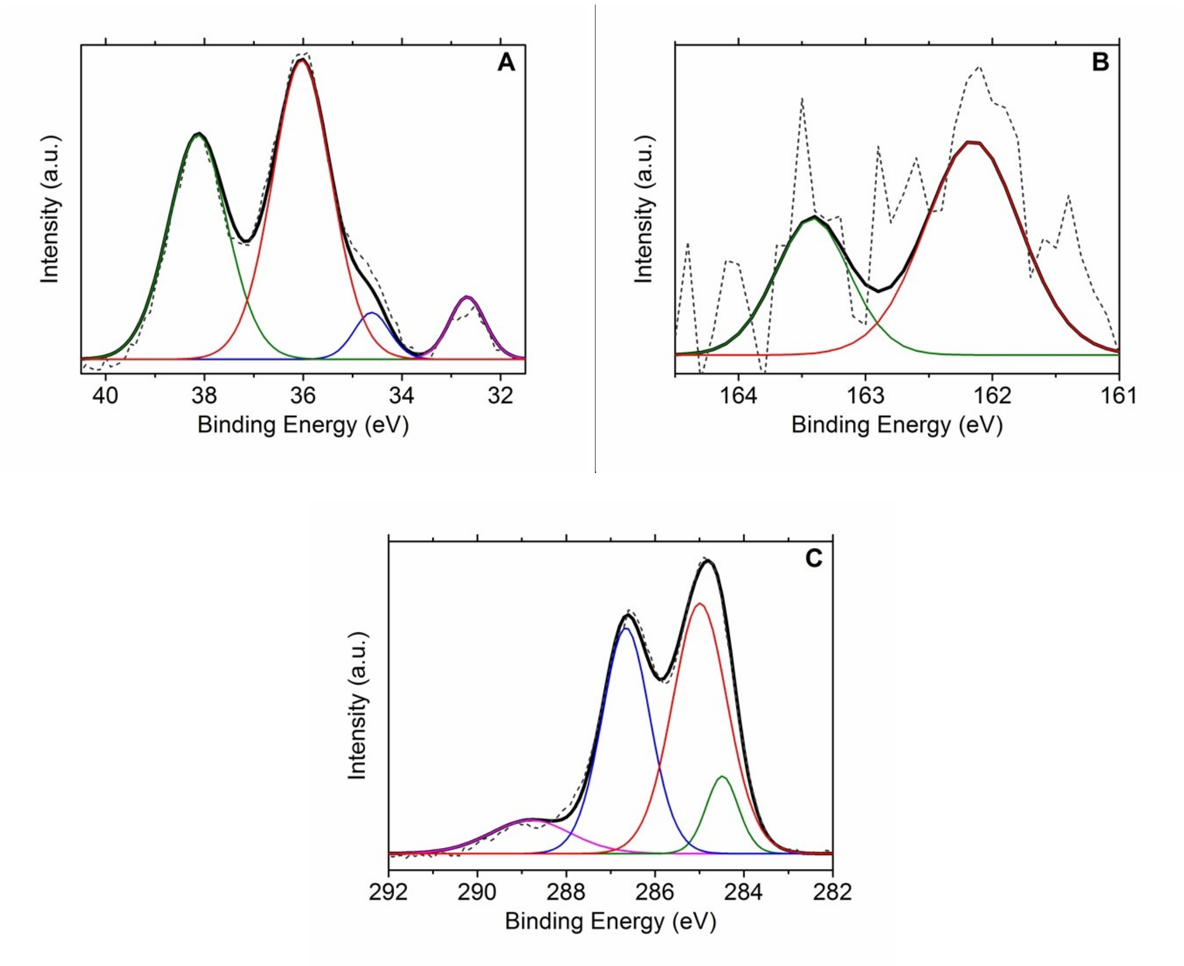

Fig. S3: **(A)** W4f XPS spectrum of a DOPC/WS_2 dispersion drop cast onto Si/SiO_2 substrate. In the fitting, the purple line corresponds to the $W^{4+}4f_{7/2}$ peak, the blue line to the $W^{4+}4f_{5/2}$ peak, the red line to the $W^{6+}4f_{7/2}$ peak and the green line to the $W^{6+}4f_{5/2}$ peak. **(B)** S2p XPS spectrum of a DOPC/WS_2 dispersion drop cast onto Si/SiO_2 substrate. The fitting is for the S2p doublet, with the red line depicting the $S2p_{3/2}$ peak and the green line the $S2p_{1/2}$ peak. **(C)** C1s XPS spectrum of a DOPC/WS_2 dispersion drop cast onto Si/SiO_2 substrate. In the fitting, the green line corresponds to the C-C sp^2 peak, the red line to the C-C sp^3 peak, the blue line to the C-O/C-N peak and the purple line to the O-C=O peak.

XPS: TiS_2

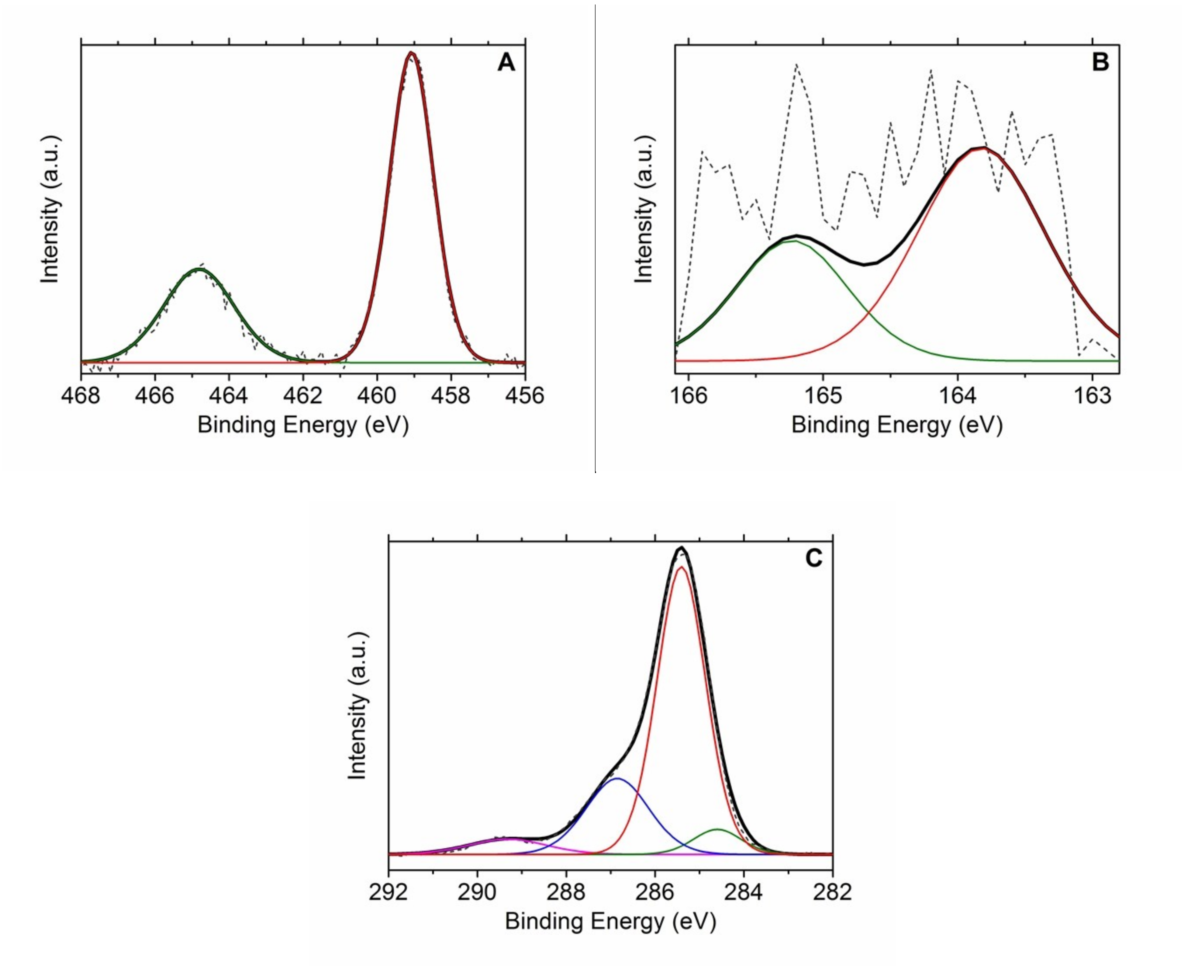

Fig. S4: **(A)** Ti2p XPS spectrum of a DOPC/TiS_2 dispersion drop cast onto Si/SiO_2 substrate. In the fitting, the red line corresponds to the $Ti2p_{3/2}$ peak and the green line to the $Ti2p_{1/2}$ peak. **(B)** S2p XPS spectrum of a DOPC/TiS_2 dispersion drop cast onto Si/SiO_2 substrate. The fitting is for the S2p doublet, with the red line depicting the $S2p_{3/2}$ peak and the green line the $S2p_{1/2}$ peak. **(C)** C1s XPS spectrum of a DOPC/TiS_2 dispersion drop cast onto Si/SiO_2 substrate. In the fitting, the green line corresponds to the C-C sp^2 peak, the red line to the C-C sp^3 peak, the blue line to the C-O/C-N peak and the purple line to the O-C=O peak.

Raman spectroscopy

Samples were prepared by drop casting 100 µL of fresh dispersions onto 1 cm^2 Si/SiO_2 substrates (pre-cleaned by sonication in ethanol and acetone for 10 min), followed by drying at 70 °C for approximately 10 min. Powder samples were used to obtain spectra for the bulk starting-materials. Raman spectra and photoluminescence (PL) measurements were taken on a Renishaw inVia system using 532 nm (2.33 eV) excitation energy (laser power of 1.8 mW) and a 100× objective (numerical aperture of 0.9), giving a spot size of approximately of 1 µm. This system uses a high-resolution grating of 1800 g mm^{-1}, giving a spectral resolution ≤1 cm^{-1}. Spectra represent three accumulations at 5 s of exposure time.

<u>Raman and PL Spectra: MoS_2</u>

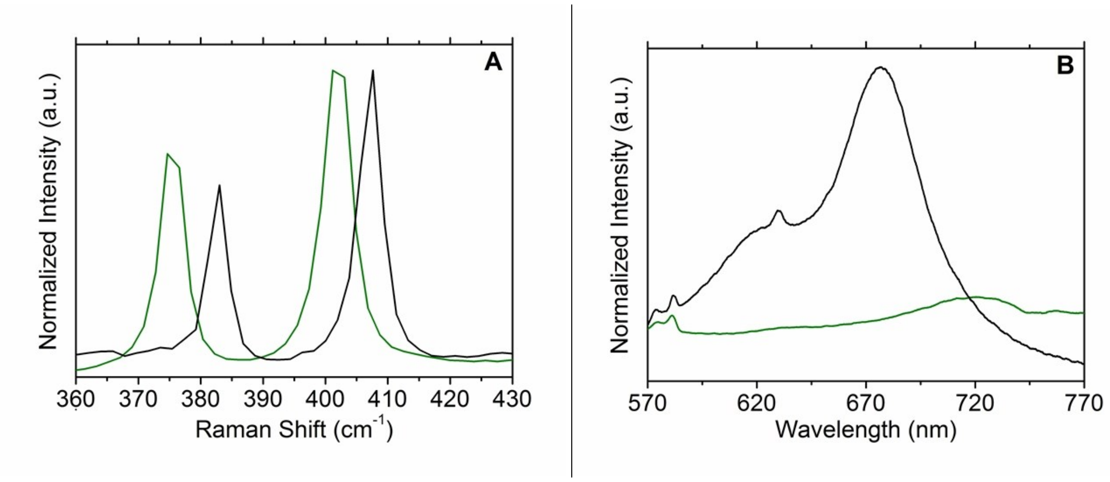

Fig. S5: **(A)** Raman spectra of the DOPC/MoS_2 dispersion drop cast onto Si/SiO_2 substrate (black line) and the bulk (powder) starting-material (green line); normalized to the A_{1g} peak. **(B)** PL spectra of the DOPC/MoS_2 dispersion drop cast onto Si/SiO_2 substrate (black line) and the bulk (powder) starting-material (green line); normalized to the A_{1g} peak.

Raman and PL Spectra: WS_2

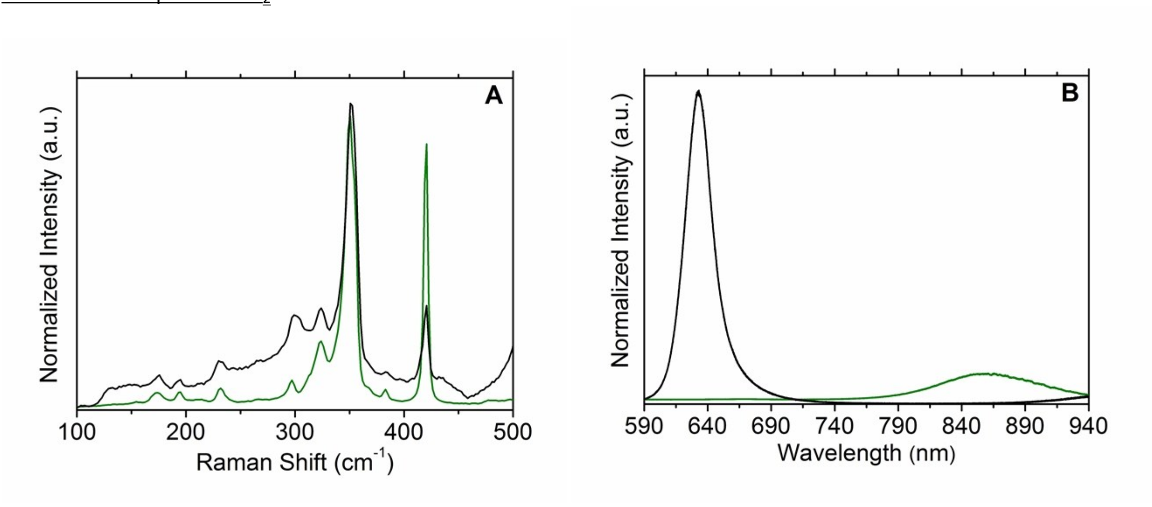

Fig. S6: **(A)** Raman spectra of the DOPC/WS_2 dispersion drop cast onto Si/SiO_2 substrate (black line) and the bulk (powder) starting-material (green line); normalized to the E^1_{2g} peak. **(B)** PL spectra of the DOPC/WS_2 dispersion drop cast onto Si/SiO_2 substrate (black line) and the bulk (powder) starting-material (green line); normalized to the E^1_{2g} peak.

Raman and PL Spectra: $MoSe_2$

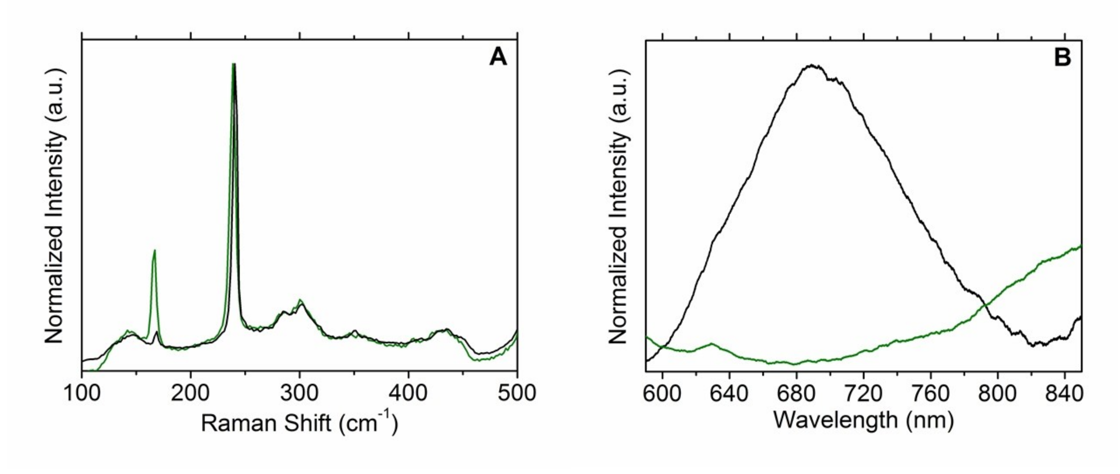

Fig. S7: **(A)** Raman spectra of the DOPC/$MoSe_2$ dispersion drop cast onto Si/SiO_2 substrate (black line) and the bulk (powder) starting-material (green line); normalized to the A_{1g} peak. **(B)** PL spectra of the DOPC/$MoSe_2$ dispersion drop cast onto Si/SiO_2 substrate (black line) and the bulk (powder) starting-material (green line); normalized to the A_{1g} peak.

Raman and PL Spectra: TiS_2

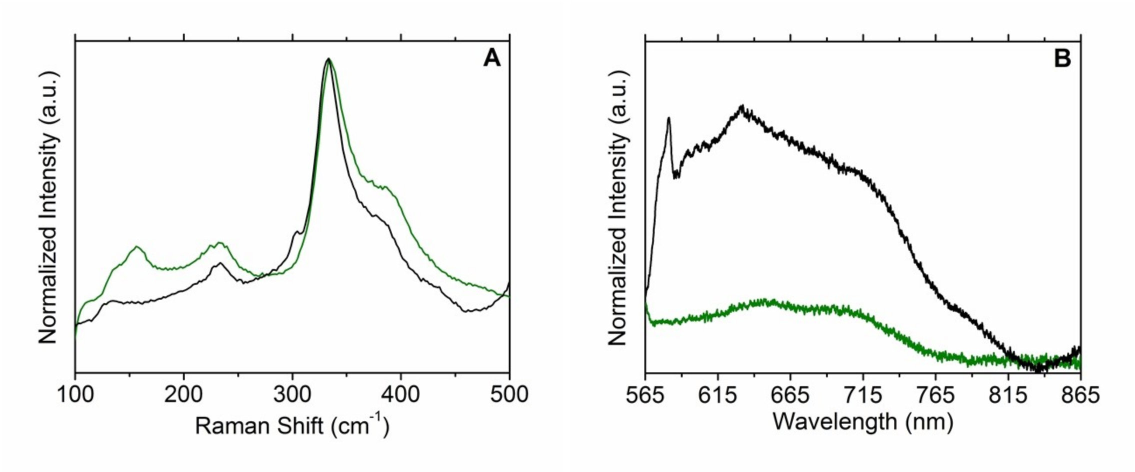

Fig. S8: **(A)** Raman spectra of the DOPC/TiS_2 dispersion drop cast onto Si/SiO_2 substrate (black line) and the bulk (powder) starting-material (green line); normalized to the A_{1g} peak. **(B)** PL spectra of the DOPC/TiS_2 dispersion drop cast onto Si/SiO_2 substrate (black line) and the bulk (powder) starting-material (green line); normalized to the A_{1g} peak.

Atomic force microscopy (AFM)

Samples were prepared by drop casting 100 µL of fresh dispersions onto 1 cm^2 clean mica substrates, which were allowed to dry overnight. AFM measurements were performed in peak-force tapping mode using silicon nitride SNL-10 cantilevers with a Multimode 8 AFM (Bruker, UK).

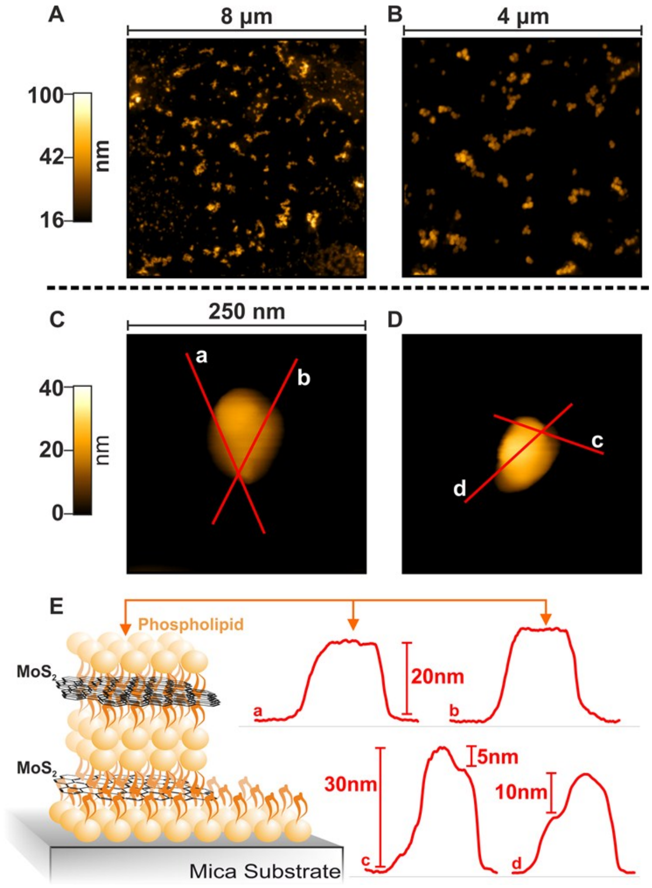

Fig. S9: Height AFM images of DOPC-coated MoS_2 for areas: 8 x 8 µm **(A)** and 4 x 4 µm **(B)**. The objected are characterised by an average height of 40 nm. Profiles acquired on single objects **(C & D)** showed “steps” of 5 nm in height (or multiples of 5 nm) **(E)** which we ascribe to a DOPC bilayer between MoS_2 sheets, as proposed in the schematic presented in **(E)**.
